# Limitations in Clinical Translation of the Age-Friendly Environment and Sarcopenia Association

**DOI:** 10.1016/j.jnha.2025.100768

**Published:** 2026-01-09

**Authors:** Yi Zou, Qi Yang, Xiaokun Tang, Mei Yang

**Affiliations:** Anyue County People’s Hospital, Ziyang 642350, Sichuan, China

To the Editor:

We read with great interest the longitudinal study by Li et al. investigating the association between an age-friendly environment (AFE), sarcopenia, and frailty among older Chinese adults [1]. The authors are commended for addressing a significant public health issue using a nationally representative cohort. Their findings, suggesting that a higher AFE score is associated with a reduced risk of incident sarcopenia and frailty, offer valuable insights for healthy aging policies. However, from a clinical and nutritional epidemiology perspective, we wish to raise critical limitations that warrant consideration for the interpretation and clinical translation of these findings.

A primary concern is the lack of objective and comprehensive nutritional assessment. Sarcopenia is profoundly influenced by protein intake, vitamin D status, and overall dietary quality [2]. The CHARLS database, as acknowledged, lacks detailed dietary data. This omission introduces substantial residual confounding, as nutritional status is a likely mediator on the causal pathway between the environment (e.g., access to markets influencing food choices) and muscle health. For instance, the counterintuitive finding that the presence of a market/convenience store increased sarcopenia risk might be partially explained by the poor nutritional quality of foods typically available in such outlets, a phenomenon documented in food environment research [3]. The absence of this data limits the mechanistic understanding of the AFE-sarcopenia relationship and hinders the development of targeted nutritional interventions alongside environmental modifications.

Furthermore, the operationalization of the AFE score may not fully capture clinical relevance. While the 35-component score is comprehensive, its clinical utility for risk stratification or guiding individual-level interventions is unclear. The study identifies specific components like "neighbor help" and "home internet" as protective, but translating these into actionable clinical advice is challenging. In clinical practice, a more parsimonious set of modifiable, evidence-based environmental factors would be more applicable. For example, the role of the physical environment in promoting physical activity is well-established for frailty prevention [4], yet the AFE score's aggregation may dilute the impact of these key elements. Future research should focus on validating a streamlined AFE tool that clinicians could use to assess a patient's environmental risk profile.

Finally, the generalizability of the AFE protective effect is limited for the most vulnerable. The subgroup analysis correctly notes that the benefit was absent in individuals with concurrent sarcopenia and frailty and those with non-independent ADL. This highlights a critical clinical gap: age-friendly initiatives, as currently measured, may be a primary prevention tool but are insufficient for managing established, complex geriatric syndromes [5]. These patients often have multimorbidity and significant functional decline, requiring integrated medical, rehabilitative, and social care models that extend beyond community infrastructure [6]. The study's findings should not be overgeneralized to suggest that environmental improvements alone can address advanced frailty.

In conclusion, while Li et al. provide crucial epidemiological evidence, future studies should incorporate robust nutritional biomarkers and dietary intake data to elucidate underlying mechanisms. We also advocate for research that develops a clinically translatable AFE screening tool and evaluates multi-domain interventions combining environmental optimization with targeted clinical care for the most frail older adults. Such steps are essential to bridge the gap between population-level findings and individualized, effective clinical and public health practice.

## Consent for publication

All authors gave their consent for publication.

## Ethics approval and consent to participate

Not applicable.

## Funding

There is no funding.

## Availability of data and materials

Not applicable.

## Declaration of competing interest

The authors declare that they have no known competing financial interests or personal relationships that could have appeared to influence the work reported in this paper.
